# The Role of Sodium in Diabetic Cardiomyopathy

**DOI:** 10.3389/fphys.2018.01473

**Published:** 2018-10-24

**Authors:** Nicolai M. Doliba, Andriy M. Babsky, Mary D. Osbakken

**Affiliations:** ^1^Department of Biochemistry and Biophysics, Institute for Diabetes, Obesity and Metabolism, School of Medicine, University of Pennsylvania, Philadelphia, PA, United States; ^2^Department of Biophysics and Bioinformatics, Ivan Franko National University of Lviv, Lviv, Ukraine; ^3^School of Biomedical Engineering, Science and Health Systems, Drexel University, Philadelphia, PA, United States

**Keywords:** sodium, calcium–sodium exchanger, NMRS, oxygen consumption, mitochondrial bioenergetics

## Abstract

Cardiovascular complications are the major cause of mortality and morbidity in diabetic patients. The changes in myocardial structure and function associated with diabetes are collectively called diabetic cardiomyopathy. Numerous molecular mechanisms have been proposed that could contribute to the development of diabetic cardiomyopathy and have been studied in various animal models of type 1 or type 2 diabetes. The current review focuses on the role of sodium (Na^+^) in diabetic cardiomyopathy and provides unique data on the linkage between Na^+^ flux and energy metabolism, studied with non-invasive ^23^Na, and ^31^P-NMR spectroscopy, polarography, and mass spectroscopy. ^23^Na NMR studies allow determination of the intracellular and extracellular Na^+^ pools by splitting the total Na^+^ peak into two resonances after the addition of a shift reagent to the perfusate. Using this technology, we found that intracellular Na^+^ is approximately two times higher in diabetic cardiomyocytes than in control possibly due to combined changes in the activity of Na^+^–K^+^ pump, Na^+^/H^+^ exchanger 1 (NHE1) and Na^+^-glucose cotransporter. We hypothesized that the increase in Na^+^ activates the mitochondrial membrane Na^+^/Ca^2+^ exchanger, which leads to a loss of intramitochondrial Ca^2+^, with a subsequent alteration in mitochondrial bioenergetics and function. Using isolated mitochondria, we showed that the addition of Na^+^ (1–10 mM) led to a dose-dependent decrease in oxidative phosphorylation and that this effect was reversed by providing extramitochondrial Ca^2+^ or by inhibiting the mitochondrial Na^+^/Ca^2+^ exchanger with diltiazem. Similar experiments with ^31^P-NMR in isolated superfused mitochondria embedded in agarose beads showed that Na^+^ (3–30 mM) led to significantly decreased ATP levels and that this effect was stronger in diabetic rats. These data suggest that in diabetic cardiomyocytes, increased Na^+^ leads to abnormalities in oxidative phosphorylation and a subsequent decrease in ATP levels. In support of these data, using ^31^P-NMR, we showed that the baseline β-ATP and phosphocreatine (PCr) were lower in diabetic cardiomyocytes than in control, suggesting that diabetic cardiomyocytes have depressed bioenergetic function. Thus, both altered intracellular Na^+^ levels and bioenergetics and their interactions may significantly contribute to the pathology of diabetic cardiomyopathy.

## Diabetic Cardiomyopathy

Diabetic cardiomyopathy is a multi-faceted disease. Diabetes is associated with an increased incidence of atherosclerotic heart disease, which results in ischemic cardiomyopathy. In addition to ischemic – heart – disease associated cardiomyopathy, there are other metabolic changes in the heart that are not necessarily related to myocardial ischemia. There is altered substrate utilization and mitochondrial dysfunction; insulin resistance; decreased flexibility in substrate use; changes in oxidative phosphorylation and the citric acid cycle; abnormalities in ketogenesis and glucose free fatty acid (FFA) cycling; and altered Ca^2+^ handling (Boudina and Abel, 2010; Veeranki et al., 2016; Jia et al., 2018).

Changes in mitochondrial morphology are associated with remodeling of the mitochondrial proteome and decreased respiratory capacity. There are changes in mitochondrial bioenergetics with decreased phosphocreatine (PCr)/ATP (shown with ^31^P NMR); decreased oxygen consumption and increased H_2_O_2_ production; defects in the ATP sensitive K^+^ channel (K_ATP_); mitochondrial uncoupling resulting in increased state 4 respiration and decreased ATP synthesis and increased oxygen consumption without increased ATP production; and mitochondrial generation of reactive oxygen species (ROS) and lipid peroxides that may activate uncoupling proteins (Boudina and Abel, 2010; Veeranki et al., 2016; Jia et al., 2018).

The combination of these various insults results in left ventricular hypertrophy, interstitial fibrosis, left ventricular diastolic and systolic dysfunction, right ventricular dysfunction, and impaired contractile reserve. The purpose of this paper is to show the importance of maintaining intracellular sodium ([Na^+^]) homeostasis in the heart and to review some of our early work of the effects of diabetes on metabolism (and vice versa) that are related to ion fluxes (Na^+^, H^+^, Ca^2+^).

## Sodium Transport Systems in Cardiomyocytes

Sodium transport processes and [Na^+^] concentration play important roles in cellular function. [Na^+^]_i_ concentration regulates Ca^2+^ cycling, contractility, metabolism, and electrical stability of the heart (Lambert et al., 2015). In the normal cell, there is a large steady-state electrochemical gradient favoring Na^+^ influx. This potential energy is used by numerous transport mechanisms, including Na^+^ channels and transporters which couple Na^+^ influx to either co- or counter-transport of other ions and solutes (Bers et al., 2003). Myocardial [Na^+^]_i_ is determined by the balance between Na^+^ influx down a trans-sarcolemmal electrochemical gradient, via Na^+^/Ca^2+^ exchanger, Na^+^/H^+^ exchanger 1 (NHE1), Na^+^/Mg^2+^ exchange, Na^+^/HCO_3_^-^ cotransport, Na^+^/K^+^/2Cl^-^ cotransport and Na^+^ channels, and Na^+^ efflux against an electrochemical gradient, mediated by Na^+^/K^+^ pump (Ottolia et al., 2013; Shattock et al., 2015). Under normal conditions, Na^+^/Ca^2+^ exchange and Na^+^ channels are the dominant Na^+^ influx pathway; however, other transporters may become important during pathological conditions. The Na^+^/Ca^2+^ exchanger transports three Na^+^ ions into the cytoplasm in exchange for one Ca^2+^ ion using the energy generated from the Na^+^ gradient as a driving force, and it is one of the main mechanisms for Na^+^ influx in cardiomyocytes (Shattock et al., 2015). The eukaryotic Na^+^/Ca^2+^ exchanger protein, as exemplified by the mammalian cardiac isoform NCX1.1, is organized into 10 transmembrane segments (TMSs; Liao et al., 2012; Ren and Philipson, 2013) and contains a large cytoplasmic loop between TMS 5 and 6 which play a regulatory role (Philipson et al., 2002). Regulation of the mammalian Na^+^/Ca^2+^ exchanger has been clearly shown both at the functional and structural levels. Allosteric regulation of the Na^+^/Ca^2+^ exchanger, by cytoplasmic Na^+^ and Ca^2+^ ions, occurs from within the large cytoplasmic loop that separates TMS 5 from TMS 6 (Philipson et al., 2002). The structures of the two regulatory domains within this region of the eukaryotic exchanger have been described (Hilge et al., 2006; Nicoll et al., 2006; Besserer et al., 2007; Wu et al., 2010). These two contiguous stretches of residues bind cytoplasmic Ca^2+^, leading to an increase in exchanger activity (Hilgemann et al., 1992; Matsuoka et al., 1995; Chaptal et al., 2009; Ottolia et al., 2009), and are identified as Ca^2+^ binding domains 1 and 2. Na^+^ ion regulation of the Na^+^/Ca^2+^ exchanger is less well studied; however, it is known that high cytoplasmic Na^+^ inactivates the exchanger (Hilgemann et al., 1992). Whether Na^+^/Ca^2+^ exchanger modulation by cytoplasmic Na^+^ is relevant to cardiac physiology remains to be established since relatively high intracellular Na^+^ concentrations (≥20 mM) are required to significantly inactivate the exchanger experimentally (Hilgemann et al., 1992; Matsuoka et al., 1995). Recently, Liu and O’Rourke (2013) revealed a novel mechanism of Na^+^/Ca^2+^ exchanger regulation by cytosolic NADH/NAD^+^ redox potential through a ROS-generating NADH-driven flavoprotein oxidase. The authors proposed that this mechanism may play key roles in Ca^2+^ homeostasis and the response to the alteration of protein kinase C (PKC) in the cytosolic pyrine nucleotide redox state during cardiovascular diseases, including ischemia–reperfusion (Liu and O’Rourke, 2013). Acting in the opposite direction, the Na^+^/K^+^ pump moves Na^+^ ions from the cytoplasm to the extracellular space against their gradient by utilizing the energy released from ATP hydrolysis. One of the strongest drivers for the activation of the Na^+^/K^+^ pump is the elevation of [Na^+^]_i_ (Shattock et al., 2015). A fine balance between the Na^+^/Ca^2+^ exchanger and the Na^+^/K^+^ pump controls the net amount of [Na^+^]_i,_ and aberrations in either of these two systems can have a large impact on cardiac function (Shattock et al., 2015). While the relevance of Ca^2+^ homeostasis in cardiac function has been extensively investigated (Ottolia et al., 2013), the role of Na^+^ regulation in heart function and metabolism is often overlooked. Small changes in the cytoplasmic Na^+^ content have multiple effects on the heart by influencing intracellular Ca^2+^ and pH levels thereby modulating heart contractility and function. Therefore, it is essential for heart cells to maintain Na^+^ homeostasis. Despite the large amount of work done in the evaluation of Na^+^ transport, there is little data that defines the metabolic support (oxidative phosphorylation, glycolysis, and ATPase activity) of Na^+^ transport under normal and pathophysiological conditions.

[Na^+^]_i_ and Na^+^ transport are altered in several diseases, including diabetes mellitus (DM) (Kjeldsen et al., 1987; Makino et al., 1987; Warley, 1991; Regan et al., 1992; Schaffer et al., 1997; Devereux et al., 2000; Hattori et al., 2000; Taegtmeyer et al., 2002; Villa-Abrille et al., 2008; Young et al., 2009; Boudina and Abel, 2010). It has been shown in heart failure myocytes, that resting [Na^+^]_i_ was increased from 5.2 ± 1.4 to 16.8 ± 3.1 mmol/L (Liu and O’Rourke, 2008). Decreased activity of the Na^+^/K^+^ pump (Greene, 1986; Kjeldsen et al., 1987; Hansen et al., 2007) and Na^+^/Ca^2+^ exchanger (Chattou et al., 1999; Hattori et al., 2000) were reported in hearts from animals with type 1 diabetes (T1DM). Many studies have also shown that the function and/ or expression of the Na^+^/K^+^ pump is reduced in cardiac hypertrophy (Pogwizd et al., 2003; Boguslavskyi et al., 2014). Previously shown, the Na^+^/Ca^2+^ exchanger protein and mRNA expression levels were significantly depressed in diabetic animal models (Makino et al., 1987; Hattori et al., 2000) and Na^+^/Ca^2+^ exchanger activity, but not mRNA, was decreased in streptozotocin-treated neonatal rats (Schaffer et al., 1997). Because the Na^+^/Ca^2+^ exchanger is the main mechanism for systolic Ca^2+^ removal, the significant reduction in exchanger activity could increase intracellular Ca^2+^ and may contribute to diabetic cardiomyopathy as a result of altered diastolic Ca^2+^ removal (Dhalla et al., 1985; Villa-Abrille et al., 2008). It has been shown that the Na^+^/Ca^2+^ exchanger activity can be restored by insulin (Villa-Abrille et al., 2008). The myocardial NHE1 was found to be enhanced in the hypertrophied Goto-Kakizaki diabetic rat heart (Darmellah et al., 2007) and led to higher [Na^+^]_i_ gain during ischemia–reperfusion (Kuo et al., 1983; Pieper et al., 1984; Pierce and Dhalla, 1985; Tanaka et al., 1992; Williams and Howard, 1994; Doliba et al., 1997; Avkiran, 1999; Xiao and Allen, 1999; Babsky et al., 2002; Anzawa et al., 2006, 2012; Williams et al., 2007). It has been suggested that elevated glucose concentrations in DM significantly influence vascular NHE1 activity via glucose induced PKC-dependent mechanisms, thereby providing a biochemical basis for increased NHE1 activity in the vascular tissues of patients with hypertension and DM (Williams and Howard, 1994). In work done by David Allen’s group, it was demonstrated that the major pathway for Na^+^ entry during ischemia appears to be the so-called persistent Na^+^ channel and the major pathway for Na^+^ entry on reperfusion is NHE1 (Xiao and Allen, 1999; Williams et al., 2007). These changes in [Na^+^]_i_ affect the Na^+^/Ca^2+^ exchanger and contribute to Ca^2+^ influx and to ROS generation, which are the major causes of ischemia/reperfusion damage (Avkiran, 1999). It has been also shown that Na^+^–glucose cotransport is enhanced in type 2 diabetes (T2DM), which increases Na^+^ influx and causes [Na^+^]_i_ overload (Lambert et al., 2015).

One of the causes of altered Na^+^ transport and increased [Na^+^]_i_ concentration can be related to the downregulation of bioenergetics. For example, in diabetes, alterations in oxidative phosphorylation may compromise ion transport (Kuo et al., 1983; Pieper et al., 1984; Pierce and Dhalla, 1985; Tanaka et al., 1992; Doliba et al., 1997). Sarcolemmal Na^+^, K^+^-ATPase function may also be depressed or down-regulated due to increased serum and intracellular fatty acids (Pieper et al., 1984). Resultant changes in intracellular cation concentrations, specifically Na^+^ and Ca^2+^, may in turn cause changes in cellular metabolism (Makino et al., 1987; Allo et al., 1991). In addition, changes in local (autocrine and paracrine) and circulating neurohormones, such as ouabain (OUA)-like (Blaustein, 1993) and natriuretic factors (Kramer et al., 1991), can exacerbate the initial changes in ion transport and result in functional abnormalities found in diabetes.

This review discusses the interdependence of Na^+^ transport and bioenergetics in the cardiac myocyte. While an energy deficit effects Na^+^ transport, on other hand, [Na^+^]_i_ has a strong effect on bioenergetics as evidenced by decreased free concentration of ATP and PCr and reduced mitochondrial respiration and oxidative phosphorylation related to changes in [Na^+^]_i_.

## Cardiomyocyte Studies in Diabetic Hearts

Dr. Osbakken’s laboratory employed unique non-invasive nuclear magnetic resonance spectroscopy (NMRS) methods for the simultaneous assessment of [Na^+^]_i_ by ^23^Na NMRS and adenine nucleotides by ^31^phosphorus (^31^P) NMRS in cardiomyocytes embedded in agarose beads (Ivanics et al., 1994; Doliba et al., 1998, 2000). ^23^Na NMRS allows for the determination of total Na^+^ signal, and [Na^+^]_i_ and extracellular Na^+^ ([Na^+^]_e_) pools by splitting into two resonances after the addition of a shift reagent to the perfusate (Doliba et al., 1998; Doliba et al., 2000; Holloway et al., 2011). This method allowed evaluation of changes in [Na^+^]_i_ in a rat model of streptozotocin-induced DM. Streptozotocin was injected intraperitoneally (60 mg/kg body wt, dissolved in citrate buffer). Myocytes were harvested four weeks after streptozotocin injection. It was found that the baseline [Na^+^]_i_ in DM cardiomyocytes increased to 0.076 ± 0.01 mmoles/mg protein (or 16.37 mmol/L) from control (Con) levels of 0.04 ± 0.01 mmoles/mg protein (or 9.3 mmol/L); *P* < 0.05 (Doliba et al., 2000). This observation is similarly reported for heart failure myocytes (Liu and O’Rourke, 2008). Of note, in DM, baseline ATP and PCr were lower compared to Con (peak area/methylene diphosphonate standard area; Doliba et al., 2000): ATP-Con: 0.67 ± 0.08, ATP-DM: 0.31 ± 0.06, *P* < 0.003; PCr-Con: 0.92 ± 0.08; PCr-DM: 0.46 ± 0.12, *P* < 0.009. This suggests that DM cardiomyocytes have depressed bioenergetics function, which may contribute to abnormal Na^+^, K^+^-ATPase function and thus result in increased [Na^+^]_i_.

**FIGURE 1:**
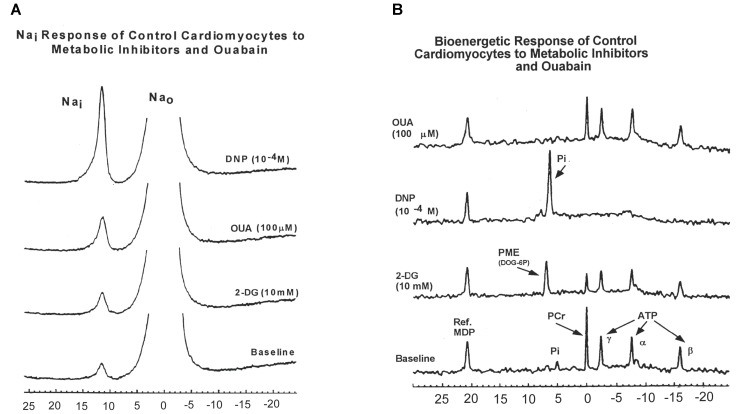
**(A)** A typical 23Na spectra obtained from control rat cardiomyocytes showing intra- and extra-cellular sodium during baseline conditions and during administration of 2-deoxyglucose (2-DG, 10 mM); 2,4-dinitrophenol (DNP, 10-4 M); and ouabain (OUA, 100 μM). **(B)** Effects of 2-DG, DNP, and OUA on ^31^P spectra obtained from control rat cardiomyocytes (typical spectra presented). MDP, methylene diphosphonate standard; PME, phosphomonoester; Pi, inorganic phosphate; PCr, phosphocreatine; ATP, adenosine triphosphate (α, γ, β); Na_i_, intracellular sodium; Na_0_, extracellular sodium. Data reprinted with permission from Doliba et al. (2000) Translated from Biokhimiya. 2000:65(4) 590-97. Copyright 2000 by MAIK “Nauka/Interperiodica”; DOI 0006-2979/00/6504-0502$25.00; Copyright permission granted by Pleiades Publishing, LLC.

To further explore these findings, we measured ^23^Na and ^31^P spectra from superfused cardiomyocytes subjected to three metabolic inhibitors: 2-deoxyglucose (2DG), 2, 4-dinitrophenol (DNP), and OUA (Figures 1A,B; Doliba et al., 2000).

Inhibition of glycolysis with 2-DG was associated with minimal or no change in [Na^+^]_i_ in DM cardiomyocytes compared to an increase in [Na^+^]_i_ in Con cardiomyocytes (DM 2DG: -4.6 ± 6%, Con 2-DG: 32.9 ± 8.1% *p* < 0.05). The Na^+^, K^+^-ATPase inhibitor, OUA, produced a smaller change from baseline in [Na^+^]_i_ in DM cardiomyocytes compared to Con (DM OUA 21.2 ± 9.2%; vs Con OUA: 50.5 ± 8.8% *p* < 0.05; Doliba et al., 2000). However, despite this apparent lower effect of OUA on DM cardiomyocytes, the absolute [Na^+^]_i_ after treatment with OUA was still 41% higher in DM cardiomyocytes compared to control due to the higher baseline [Na^+^]_i_.

In both animal models, uncoupling of oxidative phosphorylation with DNP was associated with similar large increases in [Na^+^]_i_; Con, 119.0 ± 26.9%; DM, 138.2 ± 12.6 (Figure 1A).

Figure 1B presents examples of ^31^P-NMR spectra for control cardiomyocytes obtained during baseline and 2-DG, OUA, and DNP interventions. In control cardiomyocytes, 2-DG caused a 26.4 ± 4.8% decrease of β-ATP and 35.4 ± 4.9% decrease of PCr compared to baseline. In diabetic cardiomyocytes, 2-DG caused slightly smaller decreases in β-ATP (16.2 ± 5.9%) and PCr (27.96 ± 1.7%) when compared to control. Uncoupling of oxidative phosphorylation with DNP caused apparent complete depletion (i.e., to total NMR invisibility) of both β-ATP and PCr (–100%) in both control and diabetic cardiomyocytes. The large [Na^+^]_i_ increase due to DNP intervention suggests that both groups of cardiomyocytes require oxidative ATP synthesis to support the cell membrane ion gradient.

Unexpectedly, inhibition of Na, K-ATPase with OUA produced minimal change in bioenergetic parameters in cardiomyocytes from both animal models.

In diabetic cardiomyocytes, the decreased response of [Na^+^]_i_ to OUA and 2-DG can be related to prior inhibition of Na^+^/K^+^ pump (Greene, 1986; Kjeldsen et al., 1987; Hansen et al., 2007) and glycolysis (Boden et al., 1996; Boden, 1997).

## Isolated Mitochondrial Studies in Diabetic Hearts

The [Na^+^]_i_ is tightly coupled to Ca^2+^ homeostasis and is increasingly recognized as a modulating force of cellular excitability, frequency adaptation, and cardiac contractility (Faber and Rudy, 2000; Grandi et al., 2010; Despa and Bers, 2013; Clancy et al., 2015). Mitochondrial ATP production is continually adjusted to energy demand through increases in oxidative phosphorylation and NADH production mediated by mitochondrial Ca^2+^ (Liu and O’Rourke, 2008). Mitochondria in cardiac myocytes have been recognized as a Ca^2+^ storage site, as well as functioning as energy providers that synthesize a large proportion of ATP required for maintaining heart function. In cardiac mitochondria, Ca^2+^ uptake and removal are mainly mediated via the mitochondrial Ca^2+^ uniporter and the mitochondrial Na^+^/Ca^2+^ exchanger (mNa^+^/Ca^2+^) (Gunter and Pfeiffer, 1990; Bernardi, 1999; Brookes et al., 2004; Liu and O’Rourke, 2008; Palty et al., 2010), respectively. The Ca^2+^ concentration for half-Vmax of the Ca^2+^ uniporter was estimated as ∼10–20 mM in studies of isolated mitochondria, which far exceeds cytosolic Ca^2+^ (1–3 mM; Liu and O’Rourke, 2009). By catalyzing Na^+^-dependent Ca^2+^ efflux, the putative electrogenic mNa^+^/Ca^2+^ exchanger plays a fundamental role in regulating mitochondrial Ca^2+^ homeostasis (Gunter and Gunter, 2001; Liu and O’Rourke, 2008), oxidative phosphorylation (Cox and Matlib, 1993a,b; Cox et al., 1993; Liu and O’Rourke, 2008), and Ca^2+^ crosstalk among mitochondria, cytoplasm, and the endoplasmic reticulum (ER; Szabadkai et al., 2006). The dependence of the mNa^+^/Ca^2+^ exchanger on [Na^+^]_i_ is sigmoidal with half-maximal velocity (K_0.5_) at ∼5–10 mM, which covers the range of physiological [Na^+^]_i_ in the heart (Bers et al., 2003; Saotome et al., 2005). Mitochondrial Ca^2+^ activates matrix dehydrogenases (pyruvate dehydrogenase, α-ketoglutarate dehydrogenase, and the NAD^+^-linked isocitrate dehydrogenase) (Hansford and Castro, 1985; McCormack et al., 1990; Balaban, 2002; Gunter et al., 2004) and may also activate F_0_/F_1_-ATPase (Yamada and Huzel, 1988; Territo et al., 2000, 2001), and the adenine nucleotide translocase (ANT; Moreno-Sanchez, 1985). The K_0.5_ for Ca^2+^ activation of these three dehydrogenases is in the range of 0.7–1 mM (McCormack et al., 1990; Hansford, 1991). The overall effect of elevated mitochondrial Ca^2+^ may be the upregulation of oxidative phosphorylation and the acceleration of ATP synthesis (McCormack et al., 1990; Balaban, 2002; Matsuoka et al., 2004; Jo et al., 2006). Activation of Ca^2+^-dependent dehydrogenases by Ca^2+^ increases NADH production, which is the primary electron donor of the electron transport chain. NADH/NAD^+^ potential is the driving force of oxidative phosphorylation and an increase in NADH/NAD^+^ potential leads to a linear increase of maximal respiration rate in isolated heart mitochondria (Moreno-Sanchez, 1985; Mootha et al., 1997). On the other hand, the excessive rise in mitochondrial Ca^2+^ triggers the mitochondrial permeability transition pore (PTP), resulting in pathological cell injury and death (Hajnoczky et al., 2006). Insufficient mitochondrial Ca^2+^ accumulation, secondary to cytoplasmic Na^+^ overload, decreases NAD(P)H/NAD(P)^+^ redox potential, resulting in compromised NADH supply for oxidative phosphorylation (Liu and O’Rourke, 2008). Since NADPH is required to maintain matrix antioxidant pathway flux, its oxidation causes a cellular overload of ROS (Kohlhaas and Maack, 2010; Kohlhaas et al., 2010; Liu et al., 2010; Clancy et al., 2015). ROS accumulation then contributes to oxidative modification of Ca^2+^ handling and ion channel targets to promote arrhythmias. This cascade of failures, stemming from [Na^+^]_i_ overload, is thus hypothesized to provoke triggered arrhythmias (Liu et al., 2010), which, in the context of the altered electrophysiological substrate in HF, may induce sudden cardiac death (SCD). Interestingly, chronic inhibition of the mNa^+^/Ca^2+^ exchanger during the induction of HF prevents these mitochondrial defects and abrogates cardiac decompensation and sudden death in a guinea pig model of HF/SCD (Liu et al., 2014). Therefore, the mitochondrial Ca^2+^ concentration must be kept within the proper range to maintain physiological mitochondrial function.

To further evaluate the pathophysiology of DM, our group studied mitochondrial respiratory function [state 3 and state 4 respiration, respiratory control index (RCI), ADP/O ratio, and rate of oxidative phosphorylation (ROP), using different substrates, and ion transport (calcium uptake)] in DM hearts compared to Con hearts. State 3 and RCI and ROP of DM rat heart were decreased when using pyruvate plus malate as substrates (Table 1; Doliba et al., 1997; Babsky et al., 2001). State 3 and ROP were also decreased when α-ketoglutarate was used as substrate (Table 1). The phosphorylation capacity, expressed as ADP/O ratio, appeared to be normal with both sets of substrates. The greatest decrease in substrate oxidation was observed with pyruvate, suggesting that pyruvate dehydrogenase activity is depressed in DM. It should be pointed out that in DM mitochondria, the decrease in state 3 was dependent on the concentration of pyruvate; and that the Km for pyruvate was higher in DM (0.058 ± 0.01 mM) compared to Con (0.0185 ± 0.0014), with no significant difference in V_max_ (Doliba et al., 1997). RCI was decreased approximately 35% at all pyruvate concentrations.

**Table 1:** Substrate oxidation by heart mitochondria of normal and diabetic rats.

	Rate of respiration (ng-atoms of O/min/mg protein)		Respiratory control		Rate of oxidative phosphorylation, (nmoles ADP/s/mg protein)
			
	State 3	State 4	(State 3/State 4)	ADP/O ratio	
**Pyruvate**					
Con	192.50+16.09	29.36+4.68	6.70+1.20	2.79+0.18	9.10+1.57
DM	115.29+18.15^∗^	26.44+5.01	4.40+0.54^∗^	2.74+0.24	5.07+1.42^∗^
**α-Ketoglutarate**					
Con	174.26+4.59	16.11+2.68	11.76+1.63	2.86+0.26	8.09+0.65
DM	156.81+3.45^∗^	14.28+2.83	11.58+1.86	2.71+0.18	6.58+0.20^∗^


*Mitochondria (1.2 mg of protein in mL) were prepared as described earlier (Doliba et al., 1998); respiration in state 3 was measured in the presence of 0.3 mM-ADP, and respiration in state 4 was measured after ADP was completely phosphorylated. Data reprinted by permission from Nature/Springer/Palgrave: (Doliba et al., 1997). ^∗^Significantly different from control P ≤ 0.01. Copyright 1997 by Springer Nature; License Number 4385511236859. Originally published by Plenum Press, New York 1997 (DOI 10987654321).*

**FIGURE 2:**
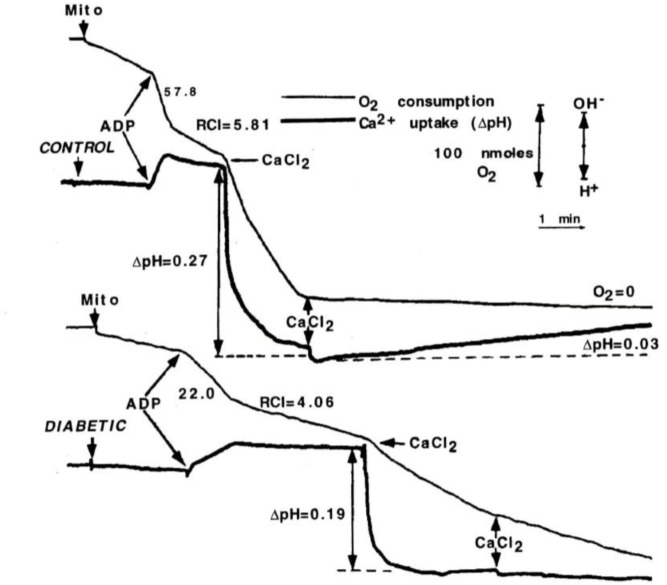
ADP and Ca^2+^-stimulated respiration of mitochondria from control and diabetic rats. Mitochondria (2 mg) were added to assay medium supplemented with 3 mM pyruvate plus 2.5 mM malate. ADP (0.3 mM) or CaCl_2_ (50 μM) was used to initiate state 3 respiration and Ca-uptake. Ca^2+^ uptake by mitochondria was monitored by using the change in H^+^ flux. Stimulation of oxygen consumption by ADP or Ca^2+^ was approximately 50% lower in DM mitochondria compared to Con. Data reprinted by permission from Nature/Springer/Palgrave: Doliba et al. (1997). Copyright 1997 by Springer Nature; License Number 4385511236859. Originally published by Plenum Press, New York 1997 (DOI 10987654321).

To determine whether changes in Ca^2+^ transport might be the cause of change in oxidative function presented above, state 3 respiration was initially stimulated by ADP, and then by CaCl_2_ in Con and DM mitochondria during pyruvate plus malate oxidation; Ca^2+^ uptake was recorded using the change in H^+^ flux (i.e., Ca^2+^/2H^+^ exchange; Figure 2; Doliba et al., 1997). Stimulation of oxygen consumption by ADP or Ca^2+^ was approximately 50% lower in DM mitochondria compared to Con. In order to measure Ca^2+^ capacity, 100 mM CaCl_2_ was added to the incubation medium and Ca^2+^ uptake was followed by changes in pH. In contrast to Con mitochondria, mitochondria from DM animals did not completely consume even the first addition of CaCl_2_. These data suggest that the Ca^2+^ capacity in heart in DM rats is greatly decreased compared to Con.

## Respiratory Function and Substrate Use Studied by Mass Spectroscopy

**FIGURE 3:**
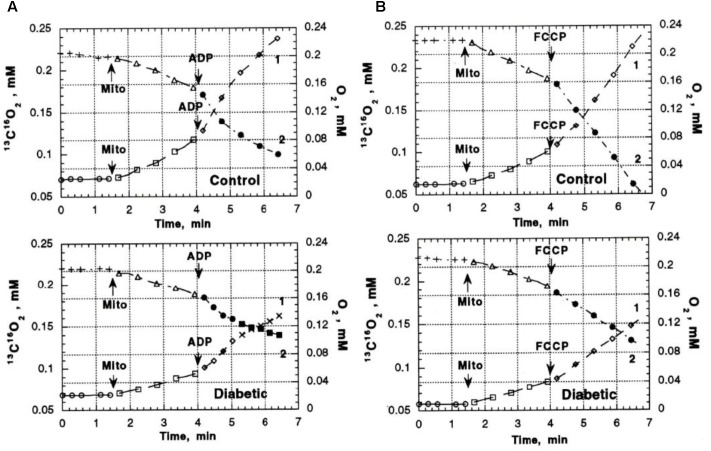
^13^C^16^O_2_ production (Boudina and Abel, 2010) and O_2_ consumption (MVO_2_) (Veeranki et al., 2016) during oxidation of [1-^13^C] pyruvate by heart mitochondria from control and diabetic rats. **(A)** ^13^C^16^O_2_ production and MVO_2_ after addition of ADP. **(B)** ^13^C^16^O_2_ production and MVO2 after addition of FCCP to uncouple oxidative phosphorylation. Both the ^13^C^16^O_2_ production and MVO_2_ stimulated by ADP FCCP were much less in DM mitochondria compared to Con. Data reprinted by permission from Nature/Springer/Palgrave: Doliba et al. (1997). Copyright 1997 by Springer Nature; License Number 4385511236859. Originally published by Plenum Press, New York 1997 (DOI 10987654321).

**FIGURE 4:**
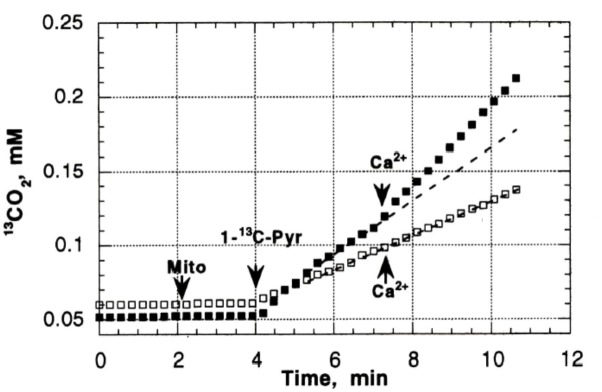
The effect of Ca^2+^ on CO_2_ production in Con (black squares) and DM mitochondria (open squares). Addition of Ca^2+^ caused minimal changes in ^13^C^16^O_2_ production in DM; whereas Ca^2+^ increased ^13^C^16^O_2_ production by 33–40% in Con. Data reprinted by permission from Nature/Springer/ Palgrave: Doliba et al. (1997). Copyright 1997 by Springer Nature; License Number 4385511236859. Originally published by Plenum Press, New York 1997 (DOI 10987654321).

Previous studies in our laboratory and laboratories of other investigators have shown abnormalities in pyruvate oxidation in animal models of DM, possibly related to effects of abnormal Ca^2+^ content on enzymes such as pyruvate dehydrogenase. To evaluate the possible role of abnormal pyruvate dehydrogenase function on respiratory function of heart mitochondria from diabetic rats, mass spectroscopy determination of O_2_ consumption and ^13^C^16^O_2_ production from [1-^13^]pyruvate were measured in heart mitochondria from Con (*n* = 8) and DM (4 weeks after streptozotocin injection; *n* = 8) rats (Doliba et al., 1997). Figure 3 presents the time course of ^13^C^16^O_2_ production (curve 1) and oxygen consumption (MVO_2_) (curve 2) during oxidation of [1-^13^C]pyruvate by heart mitochondria from Con and DM rats (Doliba et al., 1997). Both the ^13^C^16^O_2_ production and MVO_2_ stimulated by ADP (Figure 3A) or carbonilcyanide p-triflouromethoxyphenylhydrazone (FCCP), an uncoupler of respiration and oxidative phosphorylation (Figure 3B), were much less in DM mitochondria compared to Con (with ADP, 35–50% less; FCCP, 20–30% less). Addition of Ca^2+^ caused minimal changes in ^13^C^16^O_2_ production in DM; whereas Ca^2+^ increased ^13^C^16^O_2_ production by 33–40% in Con (Figure 4; Doliba et al., 1997). This lack of stimulation of a key enzyme by Ca^2+^ may be a factor in the development and progression of pathophysiological sequelae in DM and may be related to abnormal Ca^2+^ transport function. The data presented in the next two paragraphs suggest that abnormal mitochondrial Ca^2+^ transport and bioenergetics in DM cardiac mitochondria can be related to abnormalities in Na^+^ flux.

### Na^+^ Regulation of Mitochondrial Energetics: DM Modeling Effort

Previous data reported above, and by others suggest that the etiology of DM end organ damage may be related to abnormalities in Na^+^ transport. We and others (Cox and Matlib, 1993b; Cox et al., 1993; Maack et al., 2006; Liu and O’Rourke, 2008) proposed that increased [Na^+^]_i_ is involved in the regulation of mitochondrial oxidative phosphorylation through the Ca^2+^ metabolism. Mitochondrial Ca^2+^ ([Ca^2+^]_m_) plays a key role in linking ATP production to ATP demand (i.e., mechanical activity) and as Ca^2+^ rises in the cell, so does [Ca^2+^]_m_; this activates mitochondrial enzymes to step-up ATP production (Liu and O’Rourke, 2008; Kohlhaas and Maack, 2010). This relationship, which crucially matches ATP supply to demand, is blocked when [Na^+^]_i_ is elevated (Liu et al., 2010). The rise in [Na^+^]_i_ activates Na^+^/Ca^2+^ exchange in the inner mitochondrial membrane and keeps [Ca^2+^]_m_ low preventing ATP supply from meeting demand, leaving the heart metabolically compromised. Not only might this contribute to the known metabolic insufficiency in failing hearts but Kohlhaas et al. (2010) have shown that this mechanism increases mitochondrial free radical formation in failing hearts, further exacerbating injury.

**FIGURE 5:**
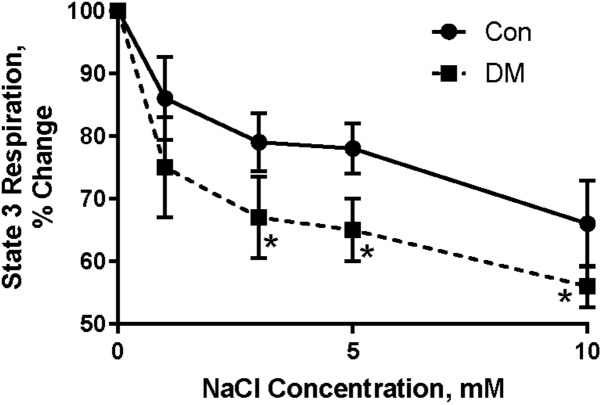
Effect of different concentrations of Na^+^ on ADP stimulated mitochondrial oxygen consumption (state 3) in control (CON) and diabetic (DM) heart mitochondria (means ± SE, *n* = 5). ^∗^ < 0.05 CON vs DM. Baseline of state 3 (without Na^+^) is assumed to be 100%. Data reprinted with permission from Babsky et al. (2001). Copyright 2001 by the Society for Experimental Biology and Medicine; DOI: 0037-9727/01/2266-0543$15.00.

To test this hypothesis, different concentrations of NaCl (in mM: 0.05; 0.1; 0.5; 1; 3; 10) were added to Con and DM mitochondria while respiratory function was monitored (Babsky et al., 2001); 1 mM a-ketoglutarate was used as substrate and mitochondrial respiration was stimulated by 200 mM ADP. Ruthenium red (1 mM), a blocker of Ca^2+^ uptake, was added to the polarographic cell before Na^+^ was added. Na^+^ in concentrations higher than 0.5–1 mM significantly decreased ADP-stimulated mitochondria oxygen consumption (Figure 5; Babsky et al., 2001). Mitochondria from DM rats were more sensitive to increasing extramitochondrial Na^+^ as demonstrated by more rapid and larger decrease in state 3 respiration (Babsky et al., 2001). The decrease in state 3 in both Con and DM mitochondria was abolished by addition of 10 mM CaCl_2_ to the polarographic cell before adding NaCl (Babsky et al., 2001). Our data agree with the studies of O’Rourke and colleagues who have shown that the elevation of [Na^+^]_i_ can impair mitochondrial energetics (Liu and O’Rourke, 2008, 2013; Kohlhaas et al., 2010; Liu et al., 2010).

### Effect of Na^+^ on Adenine Nucleotides and Pi in Con and DM Mitochondria

**FIGURE 6:**
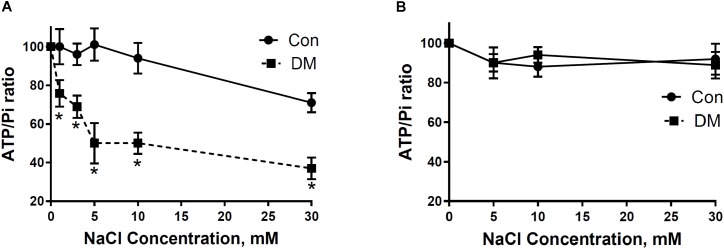
**(A)** The effect of extramitochondrial Na^+^ on ATP and Pi ratios in CON and DM heart mitochondria. **(B)** 250 μM DLTZ, an inhibitor of mitochondrial Na^+^–Ca^2+^ exchange, was added to perfusate. Baseline (without Na^+^) is assumed to be 100% (means ± SE. *n* = 4). Significance: DM vs CON: ^∗^*P* < 0.05. Data reprinted with permission from Babsky et al. (2001). Copyright 2001 by the Society for Experimental Biology and Medicine; DOI: 0037-9727/01/2266-0543$15.00.

In support of polarographic data, we used ^31^P NMRS to study the influence of different concentrations of NaCl on ATP synthesis in mitochondria isolated from Con and DM (Babsky et al., 2001). Exposure of DM mitochondria superfused at a rate of 2.7 cc/min with buffer containing Na^+^ (5–30 mM) led to greater decreases of β-ATP/Pi ratio than that found in Con (Figure 6A; Babsky et al., 2001). Diltiazem (DLTZ), an inhibitor of mitochondrial Na^+^/Ca^2+^ exchange, abolished the Na^+^ (5–30 mM) initiated decrease of β-ATP in DM mitochondria and reduced the increase of Pi with resultant values of β-ATP/Pi similar in both Con and DM mitochondria (Figure 6B; Babsky et al., 2001).

## Ischemia, Preconditioning (IPC), and the Diabetic Heart

One of most important factors of diabetic cardiomyopathy is post-ischemic myocardial injury that is associated with oxygen free radical generation, intracellular acidosis, bioenergetic depletion, as well as with abnormalities in Na^+^, H^+^, and Ca^2+^-transport in cardiomyocytes. Ca^2+^ overload and ischemic acidosis are also important intracellular alterations that could cause damage to ischemic cardiomyocytes (Bouchard et al., 2000). Sodium ions are involved in regulating both H^+^ and Ca^2+^ levels in cardiomyocytes through NHE1, Na^+^/Ca^2+^, Na^+^-K^+^-2Cl^-^, and Cl^-^/HCO_3_^-^ ion transporters. Furthermore, Na^+^ is an important regulator of bioenergetic processes in healthy and diseased cardiomyocytes (Babsky et al., 2001).

Ischemic preconditioning (IPC) is a powerful protective mechanism by which exposure to prior episodes of ischemia protects the myocardium against longer and more severe ischemic insults (Murry et al., 1986). The relationship between DM and myocardial IPC is not yet clear (Miki et al., 2012). Some studies have demonstrated that diabetes may impair IPC by producing changes in both sarcolemmal and mitochondrial K-ATP channels, which then alters mitochondrial function (Hassouna et al., 2006). These changes may lead to an elevated superoxide production which produces cellular injuries.

Ishihara et al. (2001) show in 611 patients (including 121 patients with non-insulin treated diabetes) that DM prevents the IPC effect in patients with an acute myocardial infarction. However, a study of Rezende et al. (2015) showed that T2DM was not associated with impairment in IPC in coronary artery disease patients. In fact, there is some evidence that prior short episodes of ischemia that can often occur in the diabetic heart are the substrate for IPC, whereby the heart is protected during longer episode of ischemia.

Tsang et al. (2005) hypothesized that in diabetic hearts, IPC depends on intact signaling through the phosphatidylinositol 3-kinase (PI3K)-Akt pro-survival pathway. The authors concluded that diabetic hearts are less sensitive to the IPC protective effects related to defective components in the PI3k-Akt pathway. For example, in animal models of diabetes, exposure to more prior episodes of IPC were needed to activate PI3K-Akt to a critical level and thus provide cardioprotection during exposure to longer episodes of ischemia–reperfusion than in Con.

**FIGURE 7:**
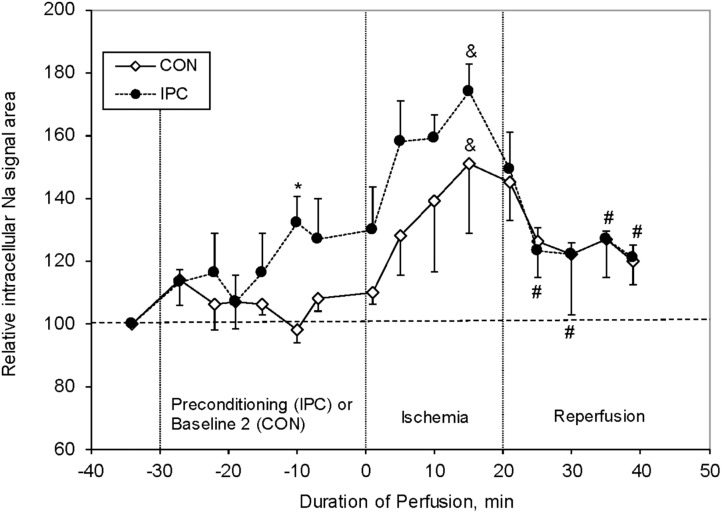
Relative changes in intracellular sodium (Na_i_) resonance areas as a function of time in control (CON, *n* = 6) and preconditioned (IPC, *n* = 4) rat hearts. Na_i_ baseline is normalized to 100. Significance: ^∗^*P* < 0.01 (IPC vs CON), ^#^*P* < 0.05 (IPC group vs end of ischemia), and ^&^*P* < 0.01 (vs pre-ischemic level for each group). Data reprinted with permission from Babsky et al. (2002). Copyright 2002 by the Society for Experimental Biology and Medicine; DOI: 1535-3702/02/2277-0520$15.00.

Our group studied the effect of IPC on [Na^+^]_i_ levels in isolated perfused rat hearts (Figure 7; Babsky et al., 2002). We have shown that 20 min ischemia increased the [Na^+^]_i_ in Con hearts by ∼50% compared to baseline. During 10–20 min of post-ischemic reperfusion the [Na^+^]_i_ significantly decreased, but was still ∼20% higher compared to baseline levels. Even though IPC significantly improved the post-ischemic recovery of cardiac function (LVDP and heart rate), unexpectedly the [Na^+^]_i_ levels were higher than Con at end IPC, and during ischemia, and were similar to Con during reperfusion. These results are in agreement with the data reported by Ramasamy et al. (1995). While our studies did not include a DM model, Ramasamy’s studies did; and showed that the % change in [Na^+^]_i_ from baseline was lower during ischemia in DM than in Con, and that the effect of the NHE1 inhibitor EIPA (similar to preconditioning ischemia) was less in DM than in Con. This suggests that the NHE1 activity was impaired in DM. The topic of NHE1 and ischemia is discussed further below.

Although diabetes mostly poses higher cardiovascular risk, the pathophysiology underlying this condition is uncertain. Moreover, though diabetes is believed to alter intracellular pathways related to myocardial protective mechanisms, it is still controversial whether diabetes may interfere with IPC, and whether this might influence clinical outcomes. We believe that ischemia developed in diabetic heart does not produce the same conditions that are developed in animal models when two–three 5-min ischemic episodes are each followed by 5–10 min of reperfusion. This difference may be a reason for the many controversies concerning relationship of IPC and the diabetic heart.

To conclude this discussion, it is likely that the changes in [Na^+^]_i_ may contribute to ischemic and reperfusion damage, possibly through their effects on Ca^2+^ overload (Allen and Xiao, 2003; Xiao and Allen, 2003; Williams et al., 2007).

## Ischemia and NHE1

Ischemic conditions may activate the NHE1. There are data that show that hyperactivity of NHE1 results of the increase in [Na^+^]_i_ that leads to Ca^2+^ overload through the Na^+^/Ca^2+^ exchanger, myocardial dysfunction, hypertrophy, apoptosis, and heart failure (Cingolani and Ennis, 2007). David Allen’s group showed that two inhibitors of NHE1, amiloride and zoniporide, cause cardioprotection which was judged by the recovery of LVDP and by the magnitude of the reperfusion contracture (Williams et al., 2007). The authors also showed that there were two different mechanisms for Na^+^ entry during ischemia and reperfusion: a major pathway for Na^+^ entry during ischemia is the persistent Na^+^ channels (I_Na,P_) and the major pathway for Na^+^ entry on reperfusion is NHE1 (Williams et al., 2007). The optimal therapy may require blocking both pathways. Pisarenko et al. (2005) show that inhibition of NHE1, similar to IPC, protects rat heart. In rabbit hearts, inhibition of NHE1 has been shown to be associated with significant protection during ischemia/reperfusion injury in immature myocardium, mostly by reducing myocardial calcium overload (Cun et al., 2007; Zhou et al., 2008). Furthermore, NHE1 inhibition leads to a decrease of infarct size after coronary artery thrombosis and thrombolysis and provides a comparable to preconditioning degree of cardioprotection against 60 min of regional ischemia (Hennan et al., 2006). NHE1 inhibition attenuates the cardiac hypertrophic response and heart failure in various experimental models. For example, early and transient administration of a NHE1 inhibitor inhibits cardiomyocyte hypertrophy in cultured cells, as well as *in vivo* cardiac hypertrophy and heart failure, suggesting a critical early NHE1-dependent initiation of hypertrophy (Kilic et al., 2014). However, in a dog model, one NHE1 inhibitor such as EMD 87580 did not protect against ischemia–reperfusion injury, and no additive protection beyond preconditioning was obtained (Kingma, 2018). It appears that NHE1 activity has a biphasic effect on myocardial function. Total blockage of activity provides a beneficial effect, but overexpression also provides cardioprotection. It is important to point out that the mitochondrial K_ATP_ channel also plays an important role during ischemia and reperfusion damage (Garlid et al., 1997; Sato and Marban, 2000). The mitochondrial damage, which is in part a consequence of closure of K_ATP_ channels, can be partially reversed by mitochondrial K_ATP_ channel openers (Xiao and Allen, 2003). Combined treatment of NHE1 by Cariporide and K_ATP_ channels by diazoxide provide the most beneficial effect (Xiao and Allen, 2003).

It is interesting to note that the cardioprotective effects of the NHE1 inhibitor, Cariporide, were tested in several clinical trials to protect the heart from ischemia during coronary artery bypass surgery (CABG; Boyce et al., 2003; Mentzer et al., 2008). While Cariporide (at its highest dose of 120 mg) provided protection against all-cause mortality and myocardial infarction at day 36 and 6 months after CABG compared to placebo, there was an increased mortality in the form of cerebrovascular events. Thus, Cariporide was not further developed for clinical use as a cardioprotection agent.

## Sodium Transport Inhibitors in Treatment of Diabetic Cardiomyopathy

The NHE1 are integral membrane proteins that may have multiple activities in the heart. Nine different NHEs have been identified. NHE1 is the major isoform found in the heart, and plays an integral role in regulation if intracellular pH, Na^+^ and Ca^2+^. Aberrant regulation and over-activation of NHE1 can contribute to heart disease and appears to be involved in acute ischemia–reperfusion damage and cardiac hypertrophy. Changes in intracellular pH related to changes in NHE1 function can stimulate the Na^+^/Ca^2+^ exchanger to eliminate intracellular Na^+^ and increase intracellular Ca^2+^ (Levitsky et al., 1998; Odunewu-Aderibigbe and Fliegel, 2014).

Pharmacological overload caused by angiotensin II, endothelin-1, and a1-adrenergic agonists can enhance the activity of the NHE1, which leads to an extrusion of H^+^ and an increase in intracellular Na^+^. Inhibition of NHE1 can reverse these effects and lead to regression of myocardial hypertrophy that can produce a beneficial effect in heart failure, and can protect against ischemic injury in genetic diabetic rat and non-diabetic rat hearts. However, at present, there are no NHE1 inhibitors that have been found to be therapeutically useful in the treatment of heart disease (Ramasamy and Schaefer, 1999; Cingolani and Ennis, 2007).

More recently, during studies of newer anti-diabetic drugs on cardiac function, it was found that Na^+^-glucose exchangers used in the treatment of diabetes provided significant cardiac protection. Further investigation into the potential etiology of this protection suggests that at least one of these drugs, Empagliflozin (EMPA) may produce this affect via inhibition of NHE1. This protective effect is apparently unrelated to EMPA effect on HbA1C. In two animal models (rabbit and rat), the effect appears to be related to decreases in cytoplasmic Na^+^ and Ca^2+^ and an increase in mitochondrial Ca^2+^. It is unclear what the effects are due to in humans, but some evidence suggests that they may be similar (Baartscheer et al., 2017; Lytvyn et al., 2017; Packer, 2017; Packer et al., 2017; Bertero et al., 2018; Inzucchi et al., 2018).

## Summary

The data presented in this review paper suggest that while changes in bioenergetic function may be a cause of ion transport abnormalities, it is as likely that abnormalities of ion content and transport may contribute to metabolic (bioenergetics and respiratory function) abnormalities. The results also suggest that increased [Na^+^]_i_ concentration in DM cardiomyocytes may be a factor, leading to chronically decreased myocardial bioenergetics. Further studies in this area may provide insight into some possible cellular and mitochondrial mechanisms which contribute to progressive pathophysiological processes as disease progresses and may set the stage for better therapies in future.

## Author Contributions

ND contributed to five sections related to sodium transport and cellular and mitochondrial bioenergetics, AB to “Effect of Sodium on Adenine Nucleotides and Pi”, AB and MO to “Ischemia and NHE1” and “Ischemia, Preconditioning and the Diabetic Heart”, MO to “Diabetes Cardiomyopathy” and “Sodium Transport Inhibitors in Treatment of Diabetic Cardiomyopathy.”

## Conflict of Interest Statement

The authors declare that the research was conducted in the absence of any commercial or financial relationships that could be construed as a potential conflict of interest.
